# Hybridization, cryptic diversity, and invasiveness in introduced variable-leaf watermilfoil

**DOI:** 10.1111/j.1752-4571.2012.00267.x

**Published:** 2012-05-10

**Authors:** Hannah F Tavalire, Gregory E Bugbee, Elizabeth A LaRue, Ryan A Thum

**Affiliations:** 1 Grand Valley State University, Annis Water Resources InstituteMuskegon, MI, USA; 2 Department of Soil & Water, Connecticut Agricultural Experiment StationNew Haven, CT, USA

**Keywords:** amplified fragment length polymorphisms, cryptic invasion, heterosis, *Myriophyllum*

## Abstract

Hybridization may be important in the evolution of invasiveness, but few empirical studies compare introduced hybrid and parental lineages. Invasive ‘variable-leaf watermilfoil’ (*Myriophyllum heterophyllum*) in the northeastern United States consists of at least three distinct lineages: an interspecific hybrid (*M. heterophyllum × Myriophyllum laxum*) and two historically allopatric lineages of pure *M. heterophyllum*. Previous observations suggested that hybrid populations of variable-leaf watermilfoil may be comparatively more ‘invasive’ than pure lineages. However, no quantitative data comparing hybrid and parental lineages have been collected, nor has invasiveness been compared between parental lineages. Here, we demonstrate that these distinct lineages are also ecologically distinct. We find some support for the hypothesis that hybridization has played a role in the evolution of invasiveness: hybrids exhibited higher biomass, individual plant size, and greater branching than at least one parental lineage of *M. heterophyllum*. However, parental lineages did not differ from the hybrid for some traits, demonstrating that pure parental lineages can also be invasive. In addition, we found no evidence for a role of intraspecific hybridization in the evolution of invasiveness in these lineages of variable-leaf watermilfoil, even where they co-occurred locally. Our study suggests that distinguishing among cryptic lineages will help prioritize rapid response control efforts.

## Introduction

In the last decade, the evolution of invasiveness through hybridization has become an important focus in the study of biological invasions. Hybridization may catalyze the evolution of invasiveness by generating novel genotypes, increasing genetic variation available for selection to act upon, fixing heterosis via stabilizing mechanisms, or dumping genetic load (Ellstrand and Schierenbeck 2001). While a large number of examples can be found where hybridization preceded the emergence of successful invasions, very few studies have quantitatively compared invasive hybrids to nonhybrids where hybridization has been suspected of playing an important role in stimulating invasiveness (Vila and D'Antonio 2010; Campbell and Waser [Bibr b3]; Wolfe et al. 2011). Such comparisons are critical, because the identification of invasive hybrid genotypes does not in itself demonstrate an important role for hybridization.

Variable-leaf watermilfoil (*Myriophyllum heterophyllum* Michx.) invaded the northeastern United States in the early to mid-1900s (Les and Mehrhoff 2004) and has spread rapidly throughout the New England region, especially in the past 30 years (Thum and Lennon 2009). Genetic analyses of native and introduced populations have revealed that invasive ‘variable-leaf watermilfoil’ actually consists of a complex of at least three morphologically cryptic but genetically distinct lineages that presumably have been independently introduced (Thum et al. 2011). Moody and Les (1999) distinguished an introduced interspecific hybrid lineage, *M. heterophyllum × Myriophyllum laxum* Schuttlew ex Chapm. (hereafter ‘hybrid’), from pure *M. heterophyllum* (Moody and Les 1997). In addition, two genetically distinct introduced lineages of pure *M. heterophyllum* can be distinguished, one originating from the US Atlantic Coastal Plain and the other originating from source(s) outside of the Atlantic Coastal Plain (ACP and ‘Continental’, respectively; Thum et al. 2011).

When Moody and Les (1999) distinguished introduced hybrids from pure *M. heterophyllum* in New England, they qualitatively noted that hybrid populations were always exhibiting invasive growth characteristics, whereas pure *M. heterophyllum* populations rarely did. By ‘invasive growth characteristics’, they referred to populations as being ‘noticeably aggressive (forming dense monospecific stands) and … found primarily in localities where plants were of local management concern’. However, to date, no quantitative measures of relative invasiveness between pure *M. heterophyllum* and hybrids have been conducted to explicitly test for differences in invasiveness. Furthermore, Thum et al. (2011) distinguished two types of introduced parental *M. heterophyllum* – ACP and Continental – but it is not clear whether the two lineages differ in growth characteristics.

An understanding of whether the cryptic variable-leaf watermilfoil lineages that have invaded the northeastern United States differ in their growth characteristics has important management implications. Currently, ‘variable-leaf watermilfoil’ populations are not routinely distinguished from one another. Thus, distinguishing which lineage(s) occurs in a particular lake could help prioritize management efforts to focus control on those that are most likely to exhibit the most nuisance growth.

Here, we quantitatively compare growth characteristics of genetically distinct pure and hybrid lineages of introduced variable-leaf watermilfoil in the northeastern United States. We focus on growth characteristics that capture aspects of aquatic plant growth habits that are likely to be considered nuisance growth and therefore reflect the ‘invasive characteristics’ that Moody and Les (1999) noted. We also tested for gene flow using amplified fragment length polymorphisms (AFLPs) to confirm that our growth measurements were taken on genetically distinct taxa (hybrid, Continental, and ACP lineages) rather than on a hybrid swarm.

## Methods

### Gene flow analysis

**Figure 1:**
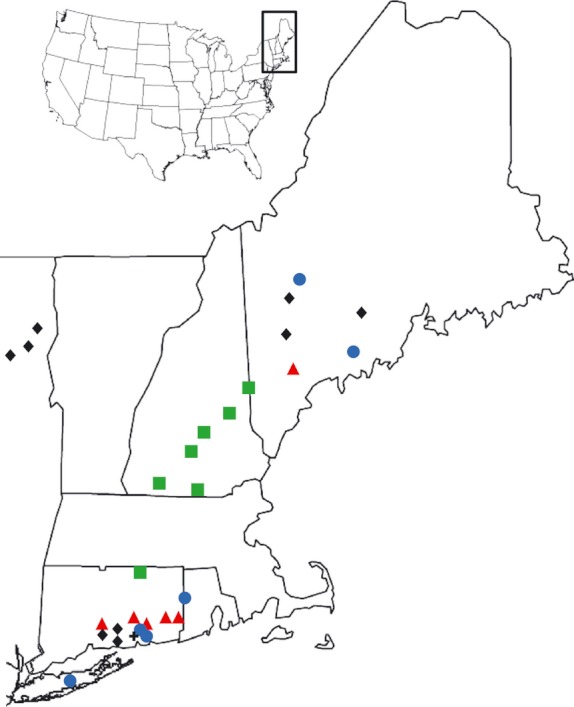
Locations of each lake used in the field observational study coded by lineage (hybrid, red triangles; continental, green squares; Atlantic Coastal Plain, blue circles; one population with both hybrid and ACP individuals is denoted by a black cross). Lakes used only for the gene flow study are denoted by black diamonds. For further information about which lineages occurred in each lake, see Table 1.

We used AFLPs to test for evidence of gene flow among invasive lineages. In total, we collected samples from 25 lakes, 14 of which are included in our growth characteristics study (see below; Fig. 1). Stems were collected from multiple locations (∼5) around each lake. We believe we were able to accurately characterize taxonomic variation within each lake using this sampling strategy for two reasons. First, the distinct genetic lineages were clearly identifiable to us as distinct morphotypes at the time of collection, and we were therefore sure to collect samples from distinct morphotypes in lakes where we found them. Second, because milfoils reproduce primarily by vegetative fragmentation (Madsen and Smith 2004), collection of one or a small number of representative individuals from a small number of sites is sufficient to distinguish lineages in each lake (Thum et al. 2011).

All molecular methods and allele scoring follow Thum et al. (2011) for a single selective primer pair (EcoRI-ACA and MseI-CAT). Here, we identified 82 polymorphic loci.

We also followed the same general procedures as Thum et al. (2011) to test for gene flow among distinct lineages using Structure v2.3.2 (Pritchard et al. 2002; Falush et al. 1969). Briefly, we used an admixture model with correlated allele frequencies, no priors, and α estimated from the data. Based on the results of Thum et al. (2011), we set the value of *K* to three to correspond to the lineages studied (i.e., hybrid, ACP, and Continental). We ran the Markov Chain for 250 000 generations preceded by a burn-in of 50 000 generations.

### Assessing nuisance growth in the field

We sampled natural populations of each of three distinct genetic lineages identified by Thum et al. (2011): ‘Continental’, ‘northeastern Atlantic Coastal Plain’, and ‘hybrid’ (*M. heterophyllum × M. laxum*). In total, we selected seven study lakes for each of the three invasive lineages, including one lake that contained two lineages (total *n* = 20 lakes; there were two study lakes where two lineages were present, but the second lineage was very rare in one of these and so we did not collect growth data on it). Study lakes encompass the geographic breadth of each lineage in the northeastern United States (Table 1 and Fig. 1). All sampling took place during June and July of 2010. To avoid potential biases in growth variables based on sampling date, we sampled lakes on a lineage-based rotation such that no single lineage was sampled exclusively early or late in the growing season and sampling date had no statistical effects on our measurements.

**Table 1:** The names and locations of all of the lakes used in each study. Inclusion in each study is noted in the ‘Study’ column (AFLP, gene flow study; Field, field observational study). Latitude and Longitude are displayed in decimal degrees

Code	Lake name	Latitude and longitude	Lineage	Study
CT 2.25	Lake Pattaganset	41.3748, −72.2338	ACP, HYB	AFLP, Field
CT 2.26	Gardner Lake	41.5141, −72.2329	HYB	AFLP, Field
CT 2.28	Amos Lake	41.5203, −71.9803	HYB	Field
CT 2.44	Black Pond	41.5275, −72.7433	HYB	AFLP, Field
CT 2.47	Gorton Pond	41.3402, −72.2099	ACP	AFLP, Field
CT 2.48	Billings Lake	41.5056, −71.8732	HYB	AFLP, Field
CT 2.50	Cedar Lake	41.4052, −72.5018	ACP	AFLP
CT 2.5	Pickerel Lake	41.5334, −72.4210	HYB	AFLP, Field
CT 2.51	Messerschmidt Pond	41.3386, −72.4902	ACP, CON	AFLP
CT 2.54	Lower Moodus Reservoir	41.5139, −72.4267	ACP, CON,HYB	AFLP
CT 2.55	Crystal Lake	41.9404, −72.3758	CON	AFLP, Field
CT 2.57	Powers Lake	41.3934, −72.2563	ACP, CON	AFLP, Field
CT 2.6	Lake Quonnipaug	41.3964, −72.6959	ACP	AFLP
ME 001	Collins Pond	43.8303, −70.4267	HYB	Field
ME 002	Shagg Pond	44.4230, −70.5320	ACP	AFLP
ME 005	Thompson Lake	44.0660, −70.4880	ACP	AFLP
ME 009	Messalonskee Lake	44.4790, −69.7890	ACP, CON	AFLP
ME 107	Bryant Pond	44.6476, −70.3780	ACP	Field
ME 110	Pleasant Pond	44.2200, −69.7890	ACP	Field
NH 005	Balch Pond	43.6167, −70.9834	CON	AFLP, Field
NH 012	Brindle Pond	43.3667, −71.2335	CON	AFLP, Field
NH 033	Turtle Pond	43.2501, −71.5167	CON	AFLP, Field
NH 303	Contoocook Lake	42.7915, −71.5167	CON	AFLP, Field
NH 304	Flints Pond	42.7915, −71.5500	CON	AFLP, Field
NH 306	Gorham Pond	43.0700, −71.6300	CON	AFLP, Field
NY 001	Long Lake	44.0150, −74.6430	CON	AFLP
NY 002	Raquette Lake	43.8410, −74.6430	CON	AFLP
NY 004	Lower Yaphank Lake	40.8420, −72.9359	ACP	AFLP, Field
NY102	Lake Flower	44.3161, −74.1203	CON	AFLP
RI 001	Carbuncle Pond	41.6986, −71.7747	ACP	Field

AFLP, amplified fragment length polymorphism.

We measured several variables related to plant growth (see descriptions below) that were intended to capture features that reflect the perception of ‘nuisance growth’ by lake residents and managers. We recognize that the evaluation of ‘nuisance growth’ is subjective to a certain degree because it can reflect different individual values and opinions. Nevertheless, we believe our variables reflect tangible aspects of plant growth that capture ‘invasiveness’ in both ecological and management contexts. Below, we give a brief description of each variable and their measurements, including an explanation for how it may reflect ‘nuisance growth’.

The percent of the littoral zone occupied captures one feature of nuisance growth. Specifically, plants that occur more widely across a lake are more likely to be considered nuisance because they potentially affect a larger fraction of the lake. We mapped the distribution of variable-leaf watermilfoil beds in each lake using Trimble GeoXT® (Trimble Navigation Limited, Sunnyvale, CA, USA) global positioning systems (GPS) with submeter accuracy. We then projected all Trimble polygons into ArcView 3.3 (Esri, Redlands, CA, USA). To calculate the percent of the littoral zone occupied by each lineage, we compared our polygons to existing bathymetric layers for each lake. Using our projected maps, we defined the littoral zone as the area within a lake that had a water depth of 4 m or less. All bathymetric layers were hand-corrected to 4 m of depth. We then overlaid our polygons on these corrected bathymetric layers and calculated the difference in areas using the AreaReturn function in ArcView 3.3. We recognize that defining the littoral zone as 4 m or less is somewhat subjective, because the depth of the actual littoral zones of lakes may depend on multiple factors. However, we believe this definition provided a logical way to standardize this measure across lakes. To assure we did not miss plant beds in more turbid lakes, we conducted rake tosses and submerged visual checks for plants in up to 4 m of water.

For the remaining growth variables (see below), we set up a 25-m transect in each of the three areas where watermilfoil was visually determined to be the densest based on the littoral zone mapping (above). For each transect, we counted the number of individual plant rosettes rooted to the substrate within a 1-m width by snorkeling with a meter stick. Because it can often be difficult to distinguish between individual plants that have underground rhizomes, rosettes were used to standardize plant counts. These counts were used in the calculations of density and biomass (below). For six transects, the water was too shallow to snorkel; for these lakes, rosettes were counted from the bow of the boat. All counts were performed by HFT to ensure consistency among lakes and transects.

We defined ‘biomass’ (g m^−3^) as the amount of plant matter present in one cubic meter of water in each bed. We calculated biomass by multiplying the average plant mass for each transect by the average number of plants per meter for that transect (total plants divided by length of transect), then divided that product by the depth of the transect ((density × average individual mass)/depth of transect). Biomass captures a second – and probably the most intuitive – feature of nuisance growth. Specifically, biomass captures the actual amount of plant material in a volume of water, and a lineage that has more biomass per unit volume is likely to be considered more of a nuisance compared to other lineages. However, note that because biomass is a function of both the number of plants and their individual mass (see below), high biomass may reflect relatively fewer plants with high individual mass or relatively more plants with lower individual mass. Thus, lineages may have different growth characteristics of individual plants but effectively exhibit the same degree of nuisance growth.

To explore the potential for such differences among lineages, we also measured ‘density’ and ‘individual mass’. We defined density as the number of individuals per square meter (i.e., calculated by dividing the total number of plants in each transect by the transect length (25 m). We measured the individual mass of three representative plants harvested from each transect. Plants were carefully dug up to obtain all of the above-ground and below-ground biomass (i.e., all roots and shoots). Each plant was individually bagged, stored in a cooler, and processed on the same day as collection. Plants were gently washed to remove periphyton and sediment. We separated above- and below-ground plant material and dried them separately in an incubator at 70°C for 24 h before weighing to ensure each plant had completely dried (based on Connecticut Agricultural Experiment Station protocols). Because we could not harvest all below-ground plant material for certain individuals, our analyses are limited to above-ground mass.

Finally, we determined the branches per unit dry mass using the same three plants used to quantify individual mass. Branching rate may capture other features of nuisance growth. For example, plants with more branches may have higher potential for spread via asexual fragmentation or plants with more branches may be more likely to form nuisance mats at the water's surface. We counted the number of terminal branches, defined as a branch with an apical meristem (i.e., a ‘tip’ on the plant). Branching rate was then defined and calculated as the number of terminal branches divided by the plant's dry mass.

### Environmental variables

Common limnological parameters were also measured at each transect and used as covariates in the analysis of each growth variable. Specifically, we recorded pH, dissolved oxygen (DO), oxidation reduction potential, temperature, and conductivity in the middle of each transect using a YSI (model 6920). Sulfuric acid titration was used to determine alkalinity in duplicate using a Hach test (model AL-TA).

### Statistical analyses

We used nested analysis of variance (anova) to test for significant differences among lineages for each of the growth variables. Akaike's information criterion (AIC) was used to select environmental covariates in each model (Table S1). For all growth variables, factors in the model included lineage, lake nested within lineage, and any environmental covariates chosen through AIC backward selection. Data were tested for normality using a Shapiro–Wilks test, and non-normal data were natural log transformed. We used orthogonal contrasts to compare the hybrid lineage to both pure lineages separately. These comparisons address how the hybrid differs in nuisance growth when compared to each lineage, while accounting for environmental covariates that paired comparisons cannot address. We also performed Holms corrected pairwise comparisons among all lineages (α = 0.05/3 = 0.017; reported in Fig. 3). All statistical tests were run in R 2.14.1 (R Development Core Team 2000).

## Results

### Gene flow

**Figure 2:**
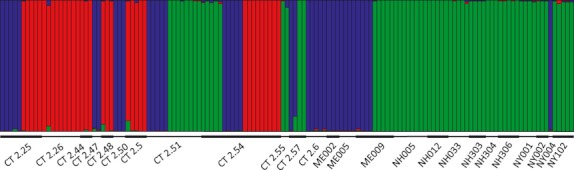
Results of structure analysis at *K* = 3. Each vertical bar corresponds to an individual, and its proportion of membership (q) to each of the three groups are indicated by different colors and correspond to previously identified genetically distinct lineages introduced to New England (Hybrid, red; Atlantic Coastal Plain, blue; Continental, green; see Thum et al. 2011). Individuals are sorted by the population of origin, shown along the *x*-axis as alternating thick and thin lines. Population codes are given in Table 1.

Not surprisingly, we found the same genetic groupings as Thum et al. (2011). Briefly, at *K* = 3, genetic groups determined in STRUCTURE correspond to the ACP, Continental, and hybrid lineages (Fig. 2).

We did not find evidence for gene flow among any of the lineages identified in our analysis. All but one individual was assigned to one of the three genetic groups with a probability of >90%, even for the 5 lakes where two or more of these lineages co-occurred. The one exception was CT 2.7 where the q value (i.e., assignment value) was slightly below 90% for the ACP group and slightly above 10% for the Continental group. In addition, we calculated confidence intervals for the assignment values of each individual to each group (using the ANCESTDIST option in Structure). The confidence intervals for all samples included 1 for the group with the highest assignment value and 0 for the other two groups. For example, even the one sample (CT 2.7) with an assignment value <90%, the confidence interval for the ACP group contained 100%, whereas the confidence interval for the Continental group contained 0.

### Nuisance growth in the field

**Figure 3:**
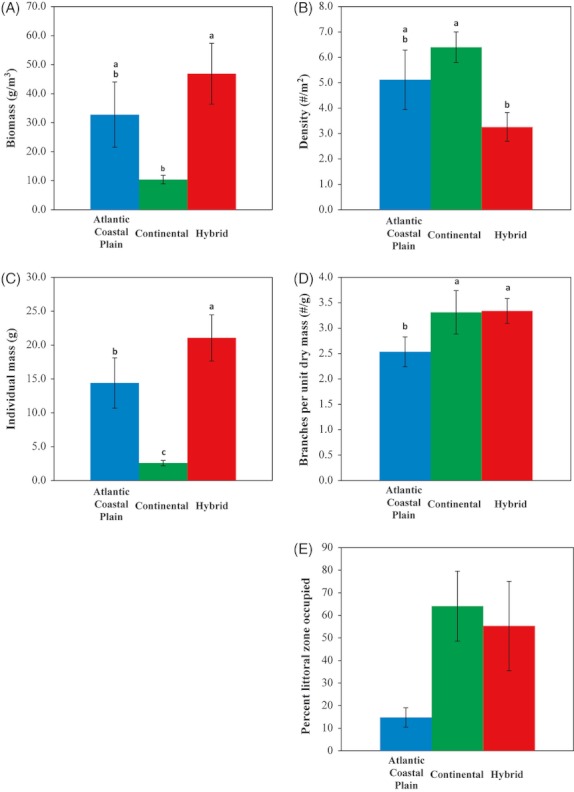
Averages of each growth variable measured: biomass (A), density (B), individual mass (C), branches per unit dry mass (D), and percent of the littoral zone occupied (E). Lineages are labeled by color (Atlantic Coastal Plain, blue; continental, green; hybrid, red). Error bars represent standard error, and significant pairwise comparisons are denoted with different letters above bars.

The primary goal of this study was to quantitatively compare growth characteristics of distinct pure and hybrid lineages of invasive variable-leaf watermilfoil in the northeastern United States. We observed a significant overall effect of lineage on all growth variables except the percent of the littoral zone occupied (Table 2). Furthermore, lineage had a medium to large conventional effect size (based on classifications in Cohen [Bibr b4]), for all other growth characteristics. Thus, it is clear that the different lineages exhibit differences in growth characteristics. Specifically, the median biomass (plant mass per meter^3^) of hybrid plants was 1.82 times larger than that of the Continental lineage (with a 95% confidence interval of 1.27–2.61), but hybrids and ACP plants were not significantly different (Table 3 and Fig. 3A; lineage effect size = 0.555; power = 0.464). There was no difference between the hybrid and ACP lineages in density, but the Continental lineage was significantly denser than the hybrid and ACP lineages (Table 3 and Fig. 3B; lineage effect size = 0.515; power = 0.407). Density negatively correlates with individual size, resulting in the fewest plants per meter^2^ in the hybrid beds, intermediate densities in the ACP beds, and the highest median densities in Continental beds by a factor of 1.58 (95% confidence interval: 1.21–2.07). All lineages differed significantly in individual mass; the hybrid and ACP plants were significantly larger than the Continental plants, but were not significantly different from each other (Table 3 and Fig. 3C; lineage effect = 1.03; power = 0.979). Contrasts revealed a 2.86-fold difference in median dry mass between the hybrid and Continental lineages (95% confidence interval: 2.41–3.40). The hybrid and Continental lineages branched significantly more per unit mass than the ACP plants by a factor of 1.28 (Table 3 and Fig. 3D; lineage effect = 0.381; power = 0.241; 95% confidence interval: 1.08–1.52), but were not significantly different from each other. Finally, there were no significant differences among any lineages for the percent littoral zone occupied (Table 3 and Fig. 3E).

**Table 2:** anova tables for all growth parameters measured including environmental covariates. Nesting variables appear inside parentheses. Significance was assessed at the *P* = 0.05 level

	df	SS	MS	*F*	*P*-value
Logit (percent of littoral zone occupied)					
Lineage	2	3.942	1.971	0.980	0.403
Error	12	24.125	2.010		
Biomass					
Lineage	2	14.554	7.277	9.695	<0.001
ln(Alkalinity)	1	2.338	2.338	3.115	0.087
ln(Conductivity)	1	0.905	0.905	1.205	0.280
ln(pH)	1	3.703	3.703	4.933	0.033
Lake(Lineage)	15	15.470	1.031	1.374	0.217
Error	33	24.770	0.751		
ln(Density)					
Lineage	2	5.873	2.936	11.525	<0.001
Temperature	1	0.019	0.019	0.076	0.784
ORP	1	0.575	0.575	2.255	0.142
Lake(Lineage)	15	12.882	0.859	3.371	0.002
Error	34	8.662	0.255		
ln(Individual Dry Mass)					
Lineage	2	41.999	20.999	55.637	<0.001
DO	1	0.080	0.080	0.212	0.648
ln(Alkalinity)	1	2.959	2.959	7.840	0.008
ln(pH)	1	0.538	0.538	1.425	0.240
Lake (Lineage)	18	21.710	1.206	3.196	0.001
Error	38	14.343	0.377		
ln(Branching Rate Per Unit Dry Mass)					
Lineage	2	1.908	0.954	7.704	0.002
ln(Alkalinity)	1	0.281	0.281	2.268	0.140
Lake (Lineage)	19	7.932	0.418	3.371	0.001
Error	39	4.830	0.124		

DO, dissolved oxygen; ORP, oxidation reduction potential.

**Table 3:** Summary of contrasts for all growth variables measured in the field. When significant, the lineage in bold had the larger value for that given measurement. The estimate describes the difference in each trait between lineages. Significance was evaluated at the *P* = 0.05 level

Measurement	Contrast	Estimate	*P*-value
Logit(Percent of Littoral Zone Occupied)	Hybrid vs ACP	0.662	0.225
	Hybrid vs Continental	−0.587	0.280
ln(Biomass)	Hybrid vs ACP	−0.002	0.993
	**Hybrid** vs Continental	0.599	0.002
ln(Density)	Hybrid vs ACP	0.073	0.589
	Hybrid vs **Continental**	−0.458	0.001
ln(Individual Dry Mass)	Hybrid vs ACP	−0.258	0.09
	**Hybrid** vs Continental	1.052	<0.001
ln(Branching Rate per Unit Dry Mass)	**Hybrid** vs ACP	0.246	0.006
	Hybrid vs Continental	−0.143	0.435

The bolded lineage displayed higher values for the given trait in a significant contrast.

In addition to differences among lineages, we also found evidence that environmental factors impact growth characteristics. Environmental covariates for each growth variable were selected using AIC (Table S1), and at least one environmental variable was included in the linear model for each growth variable, except for the percent of the littoral zone occupied. In the final models, alkalinity was found to significantly influence individual mass with a small conventional effect size (0.194), and pH significantly influenced biomass with a medium conventional effect size (0.253). All other environmental covariates included in individual models were not significant (Table 2). In addition to the specific environmental covariates, we found a large significant effect of lake nested within lineage in all models except biomass and littoral zone occupied (effect size range: 1.07–0.60). The significant among-lake variation may reflect differences in growth related to unmeasured environmental variables.

## Discussion

Previous genetic studies of variable-leaf watermilfoil have identified three distinct lineages that have been introduced to the northeastern United States, including an interspecific hybrid (Moody and Les 1999) and two distinct lineages of pure *M. heterophyllum* (Thum et al. 2011). In this study, we show that these three genetically distinct lineages also exhibit clear differences in their growth patterns. While all three lineages are local management concerns, their different growth patterns may influence the degree to, and scale in which, they are treated as ‘invasive’ populations that warrant targeting for management (e.g., herbicide applications and physical removal) to curtail negative impacts on lake ecosystem services such as water quality or recreation.

The amount of plant material in a given volume of water will have a large impact on the extent to which watermilfoil will be considered a nuisance in a lake. Watermilfoil populations with larger amounts of biomass are more likely to impact recreation and aesthetics and be targeted for control. Overall, lakes containing the hybrid lineage had the highest amount of biomass. However, while hybrid biomass was significantly higher than Continental lineage biomass, it was not significantly higher than ACP biomass. As such, water resource managers may wish to prioritize management resources to existing and new infestations of hybrid and ACP lineages. It is important to recognize that the amount of biomass that is considered a nuisance reflects decision-making processes by lake residents, associations, and managers. Thus, although the Continental lineage had the lowest biomass compared to the other two lineages, it may achieve levels of biomass within local lakes that warrant management.

Another factor that will influence the degree to which watermilfoil will be considered a nuisance in a lake is how widely distributed across the lake the plants are; all else being equal, greater distribution across the lake will be considered a greater nuisance. In our study, the three lineages did not significantly differ in the percent of the littoral zone that they occupied. However, our observations suggest that this result reflects limited power to detect differences as opposed to a lack of biological differences among lineages. For example, we observed that the ACP lineage tended to occupy shallow, sheltered areas of lakes, forming locally dense populations, but only in scattered locations throughout a lake. In contrast, the hybrid and Continental lineages tended to be more widely distributed throughout individual lakes. Both the hybrid and the Continental lineages had significantly higher branching per unit mass than the ACP lineage, which might reflect a higher capacity for asexual spread by fragmentation and would account for their more widespread presence within each lake.

In addition to the distribution of biomass within individual lakes, the total number of lakes occupied by a lineage influences the degree to which it may be targeted for management, especially by managers charged with reducing the impacts of invasive species across geographic regions that encompass multiple lakes (e.g., state or regional level biologists). Ultimately, the distribution of each lineage will be determined by its dispersal ability and the availability of lakes with suitable environmental conditions. It is unclear whether the three lineages exhibit significant differences in the environmental conditions to which they are best suited. However, it is clear that the Continental lineage is the most broadly distributed across New England (see Thum et al. 2011). It is possible that this reflects either a longer history in the region (i.e., was introduced earlier than the other two lineages and has therefore had a longer time to disperse among lakes across the region), higher dispersal capacity or opportunities, or a greater availability of lakes with suitable environmental conditions. However, it may also reflect a greater capacity for inter-lake spread. For example, the higher branching rate per unit dry mass may reflect a relatively higher investment in dispersal through asexual fragmentation in comparison with the ACP lineage.

Although it is unclear whether the lineages differ in their performance in different environmental conditions, it is clear that growth patterns are influenced by environmental variables. We found a significant lake effect for all of the individual-level growth characteristics that we measured (individual mass, density, and branching rate per unit dry mass), suggesting that local environment influences plant growth. We cannot be certain which environmental variables have the greatest influence on growth because effect sizes were very small for the limnological variables included in our model selection and linear models (e.g., pH, alkalinity, and DO), and it is possible that specific environmental variables that are important were not measured. However, it is also clear that environmental variation alone does not explain the observed variation in growth patterns, and that much of the differences that we see reflect genetic differences among the lineages. Nevertheless, further work should focus on the influence of environmental variables on the different lineages; such research might facilitate a better understanding of the local environmental conditions that facilitate local nuisance growth (e.g., biomass) for each lineage.

Interspecific hybridization is increasingly recognized as a mechanism stimulating the evolution of invasiveness (Ellstrand and Schierenbeck 2001; Schierenbeck and Ellstrand 2010). In this study, we explicitly compared introduced hybrid and parental lineages of *M. heterophyllum* and found some evidence that suggests hybridization may play an important role in the evolution of invasiveness, but is not necessarily required, in variable-leaf watermilfoil. For example, individual hybrid plants were significantly larger (i.e., had higher ‘individual mass’) than Continental plants. Similarly, stands of hybrids had significantly higher biomass per volume as compared to stands of Continental plants. However, in comparison with ACP plants, individual hybrid plants were not significantly larger nor did hybrid stands have significantly higher biomass per volume. Thus, whether or not one considers hybrid plants as more invasive than ‘pure’ *M. heterophyllum* depends on which lineage of pure *M. heterophyllum* is being compared. Nevertheless, it is still possible that hybridization played a significant role in the evolution of the particular traits exhibited by the introduced hybrid lineage. Testing this hypothesis will require comparisons of the introduced hybrid to native parental lineages of *M. heterophyllum* and *M. laxum*, whereas the focus of this study was to compare the actual introduced lineages of variable-leaf watermilfoil in the northeastern United States.

Intraspecific hybridization among previously isolated lineages may also play a role in the evolution of invasiveness (e.g.,Facon et al. 2000; Kolbe et al. 2005; Lavergne and Molofsky 2007). Certainly, the independent introduction of genetically distinct lineages into the northeastern United States provides opportunities for these previously isolated lineages to hybridize. However, we did not find any evidence for gene flow among the distinct introduced lineages in our study, even within the four lakes where two or more of them co-occurred. It is not known whether the lack of gene flow observed among the distinct lineages reflects reproductive isolating mechanisms or a lack of sufficient time for hybrid genotypes to develop and spread. Given the potential importance of hybridization in generating novel genotypes in invasions (Ellstrand and Schierenbeck 2001; Schierenbeck and Ellstrand 2010), further genetic monitoring and studies of the isolating mechanism(s) among lineages are warranted.

## Data archiving statement

Data for this publication available in Supporting information.

## Supporting Information

**Data S1.** Gene flow AFLP data.

**Data S2.** Growth characteristics data.

**Table S1.** Steps in the backward AIC model selection for each transect-level response variable.
