# Supplementary material for: Hybridization, cryptic diversity, and invasiveness in introduced variable-leaf watermilfoil
Source: Evol Appl. 2012 May 10;5(8):892–900. doi: 10.1111/j.1752-4571.2012.00267.x (PMC3552406; doi:10.1111/j.1752-4571.2012.00267.x)
Supplement: Supplementary file 2 [file eva0005-0892-SD2.doc]

| **Biomass Models** | **AIC** | **ΔAIC** | **K** |
| --- | --- | --- | --- |
| ln(Biomass) ~ Lineage + ln(Alk) + ln(Cond) + Lake(Lineage)* | -3.88 | 0 | 5 |
| ln(Biomass) ~ Lineage + ln(Alk) + ln(Cond)+ln(pH) + Lake(Lineage) | -2.9 | 0.98 | 6 |
| ln(Biomass) ~ Lineage + Temp+ln(Alk) +ln(Cond)+ ORP + Lake(Lineage) | -1.16 | 2.72 | 7 |
| ln(Biomass) ~ Lineage + DO + Temp+ln(Alk) + ln(Cond) + ORP + Lake(Lineage) | 0.76 | 4.64 | 8 |
| ln(Biomass) ~ Lineage + DO + Temp + ln(Alk) + ln(Cond) + ln(pH) + ORP+ Lake(Lineage) | 3.89 | 7.77 | 9 |
|  |  |  |  |
| **Density Models** | **AIC** | **ΔAIC** | **K** |
| ln(Density) ~ Lineage + Temp+ORP + Lake(Lineage)* | -58.82 | 0 | 5 |
| ln(Density) ~ Lineage + DO+ Temp+ORP + Lake(Lineage) | -57.76 | 1.06 | 6 |
| ln(Density) ~ Lineage + DO+Temp+ln(Cond)+ ORP + Lake(Lineage) | -56.49 | 2.33 | 7 |
| ln(Density) ~ Lineage + DO + Temp+ ln(Cond) + ln(pH) + ORP + Lake(Lineage) | -54.55 | 4.27 | 8 |
| ln(Density) ~ Lineage + DO + Temp + ln(Alk) + ln(Cond) + ln(pH) + ORP+ Lake(Lineage) | -52.56 | 6.26 | 9 |
|  |  |  |  |
| **Dry Mass Models** | **AIC** | **ΔAIC** | **K** |
| ln(DM) ~ Lineage + DO + ln(Alk) + ln(pH) + Lake(Lineage)* | -42.76 | 0 | 6 |
| ln(DM) ~ Lineage + DO + ln(Alk) + ln(pH) + ORP + Lake(Lineage) | -41.24 | 1.52 | 7 |
| ln(DM) ~ Lineage + DO + ln(Alk) + ln(Cond) + ln(pH) + ORP + Lake(Lineage) | -39.49 | 3.27 | 8 |
| ln(DM) ~ Lineage + DO + Temp + ln(Alk) + ln(Cond) + ln(pH) + ORP+ Lake(Lineage) | -37.49 | 5.27 | 9 |
|  |  |  |  |
| **Branching Rate per Unit Mass Models** | **AIC** | **ΔAIC** | **K** |
| ln(Branching) ~ Lineage + ln(Alk) + Lake(Lineage)* | -112.24 | 0 | 4 |
| ln(Branching) ~ Lineage + ln(Alk) + ln(Cond) + Lake(Lineage) | -112.14 | 0.10 | 5 |
| ln(Branching) ~ Lineage + ln(Alk) + ln(Cond)+ln(pH) + Lake(Lineage) | -111.89 | 0.35 | 6 |
| ln(Branching) ~ Lineage + ln(Alk) +ln(Cond)+ ln(pH) + ORP + Lake(Lineage) | -110.31 | 1.93 | 7 |
| ln(Branching) ~ Lineage + DO + ln(Alk) + ln(Cond) + ln(pH) + ORP + Lake(Lineage) | -108.55 | 3.69 | 8 |
| ln(Branching) ~ Lineage + DO + Temp + ln(Alk) + ln(Cond) + ln(pH) + ORP+ Lake(Lineage) | -106.58 | 5.66 | 9 |

Table S1: Steps in the backward AIC model selection for each transect-level response variable. ΔAIC reports the difference between each model and the most appropriate model, and K is the number of estimable coefficients in each model, including the intercept.

*Model used in each analysis
